# Dendrites equip neurons with a range of resonant frequencies

**DOI:** 10.1186/1471-2202-13-S1-P46

**Published:** 2012-07-16

**Authors:** Jonathan Laudanski, Benjamin Torben-Nielsen, Idan Segev, Shihab Shamma

**Affiliations:** 1 Equipe Audition, Departement d’Etude Cognitive, Ecole Normale Superieure, Paris, France; 2 Edmund and Lily Safra Center for Brain Sciences, Hebrew University, Jerusalem, Israel; 3 Department of Neurobiology, Hebrew University, Jerusalem, Israel; 4 Department of Electrical & Computer engineering, University of Maryland, USA

## 

Resonance describes the ability of neurons to respond selectively to inputs at preferred frequencies [1]. When measured at the soma, neurons typically have one dominating resonant frequency to which they respond stronger than to other frequencies. A variety of ionic mechanisms support resonance and oscillation in neurons; the majority of these channels reside in the dendritic membrane. Here we analyzed the impact of low-threshold potassium current (KLVA) in dendrites, utilizing two approaches to gain insights into the role of dendrites in neuronal resonance.

First, an analytical approach was used whereby the K_LVA_ voltage-dependence was linearized and the transfer impedance of in model consisting of a soma coupled to a cylindrical cable was derived analytically for each location along the dendrite. Changing the total density of K_LVA_, gave rise to different resonant frequencies along the dendrite. This enables us to identify the membrane features that influence the range of resonances along the dendrites to characterize the trade-off between the range of frequencies and the Q-factor (i.e., the “strength” of a resonance frequency).

Second, we used a numerical approach to optimize dendritic features to create neuron models with a large range of resonant frequencies along their dendrites. We thus confirmed the analytical results and addressed the more complicated dendritic structures including branching. We found that dendritic branches (bifrucations) may increase the range of resonant frequencies in dendrites and, at least partially, may overcome the strong trade-off between resonant strength and the possible range of resonance frequencies found it unbranched structures.

We argue that the computational complexity of neurons is increased significantly by dendrites endowed with a whole range of resonant frequencies and discuss the advantage of having a bank of differential dendritic resonances that act as dynamic filters which, following plastic processes enable neurons to resonate in a particular desirable frequencies.
